# Monitoring fluid intake by commercially available smart water bottles

**DOI:** 10.1038/s41598-022-08335-5

**Published:** 2022-03-15

**Authors:** Rachel Cohen, Geoff Fernie, Atena Roshan Fekr

**Affiliations:** 1 The Kite Research Institute, Toronto Rehabilitation Institute - University Health Network, Toronto, Canada; 2 Institute of Biomedical Engineering, University of Toronto, Toronto, Canada; 3 Department of Surgery, University of Toronto, Toronto, Canada

**Keywords:** Disease prevention, Nutrition, Biomedical engineering

## Abstract

Fluid intake is important to prevent dehydration and reduce recurrent kidney stones. There has been a trend in recent years to develop tools to monitor fluid intake using “smart” products such as smart bottles. Several commercial smart bottles are available, mainly targeting health-conscious adults. To the best of our knowledge, these bottles have not been validated in the literature. This study compares four commercially available smart bottles in terms of both performance and functionality. These bottles are the H2OPal, HidrateSpark Steel, HidrateSpark 3, and Thermos Smart Lid. One hundred intake events for each bottle were recorded and analyzed versus ground truth obtained from a high-resolution weight scale. The H2OPal had the lowest Mean Percent Error (MPE) and was able to balance out errors throughout multiple sips. The HidrateSpark 3 provided the most consistent and reliable results, with the lowest per sip error. The MPE values for HidrateSpark bottles were further improved using linear regression, as they had more consistent individual error values. The Thermos Smart Lid provides the lowest accuracy, as the sensors do not extend through the entire bottle, leading to many missed recordings.

## Introduction

**Figure 1:**
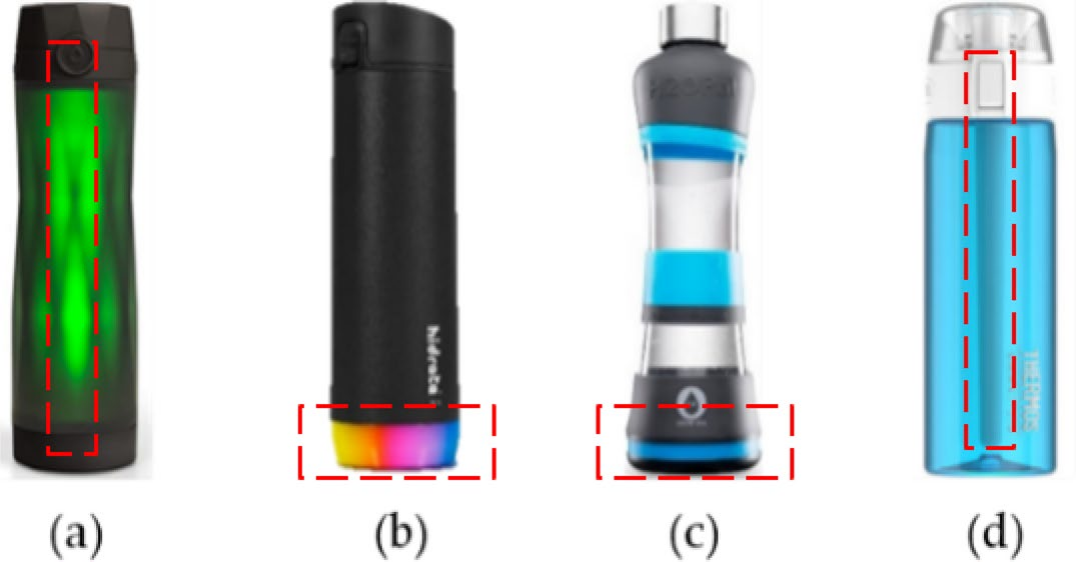
Images of analyzed commercial bottles: (**a**) HidrateSpark 3^4^, (**b**) HidrateSpark Steel^5^, (**c**) H2OPal^6^, (**d**) Thermos Smart Lid^7^. The dashed red boxes show the location of the sensors.

Dehydration is a very serious issue as it can lead to adverse complications, including confusion, falls, hospitalization, and death. Fluid intake balance is important especially among the elderly and people with underlying conditions affecting fluid regulation. High fluid intake is recommended for patients at risk of recurring stone formation. Therefore, monitoring fluid intake is a useful way to determine if adequate fluid has been consumed^1,2^. There are many reports in the literature of attempts to create systems or devices that can aide in tracking and managing fluid intake^3^. Unfortunately, most of these studies have not resulted in commercially available products. The bottles that are available in the market mainly target recreational athletes or health-conscious adults hoping to increase their hydration^3^. In this paper, we aim to determine if common commercially available water bottles are a viable solution for researchers and patients alike. We compare four commercial water bottles in terms of performance and functionality. These bottles are the HidrateSpark 3^4^, HidrateSpark Steel^5^, H2O Pal^6^ and the Thermos Smart Lid^7^, as shown in Fig. 1. These were selected as they were the only four popular bottles that were (1) available to purchase in Canada and (2) had sip volume data that could be accessed via a mobile app.

Out of the aforementioned bottles, only a previous version of the HidrateSpark has been validated in research^8^. This study found that over a 24 h fluid intake period, the HidrateSpark bottle was accurate in measuring total intake within 3% error^8^. The HidrateSpark has also been used in clinical studies to monitor intake volume in kidney stone patients^9^. Since then, HidrateSpark has developed new bottles with different sensors. The H2OPal has been used in other research studies to track and promote fluid intake, but there is no specific study to validate its performance^2,10^. Plecher et al. compared several commercial bottles in terms of functionality for seniors and available information online, however they did not perform any validation on their accuracy^11^.

All four commercial bottles include a free proprietary application to display and store intake events that are transferred via Bluetooth. The HidrateSpark 3 and the Thermos Smart Lid have the sensors down the middle of the bottle, likely using capacitive sensors, whereas the HidrateSpark Steel and H2Opal have sensors in the base, using load or pressure sensors. The sensor locations are shown in Fig. 1 by the dashed red boxes. In the Thermos Smart Lid, the sensor does not reach the bottom of the container.

## Methods

Each bottle was tested in two phases: (1) a controlled sip volume phase and (2) a free-living phase. In both phases, the results recorded by the bottle (obtained from the products’ mobile apps used on Android 11) were compared to the ground truth obtained using a 5 kg weight scale (Starfrit Electronic Kitchen Scale 93756). All bottles were calibrated before data collection using the apps. In Phase 1, sip sizes ranging from 10 to 100 mL in increments of 10 mL were measured in a random order, five times each—for a total of 50 measurements per bottle. These events were not actual drinking events by a human but were poured out so the volume of each sip could be better controlled. In this phase, the bottles were recalibrated if the sip error was larger than 50 mL and were re-paired if the app lost Bluetooth connection with the bottle. In the free-living phase, a single user drank from the bottles freely during the day, taking varying sip sizes of their choice. This phase also consisted of 50 sips over time, but not all in succession. Therefore, each bottle had a dataset of 100 measurements in total.

To determine summative liquid intake and ensure proper daily hydration, it is more important to have an accurate volume intake detection throughout the whole day (24 h), rather than of each sip. However, to determine just-in-time intervention prompts, there is a need for each sip to have a low error, as done in the study by Conroy et al.^2^. If a sip is not recorded or is poorly recorded, it is crucial that the bottle can balance out the volume in the next recordings. Therefore, the error (measured volume − actual volume) was adjusted manually. For example, assume that the subject drinks 10 mL and the bottle reports 0 mL, but then subsequently the subject drinks 20 mL and the bottle reports 30 mL total, then the adjusted error is 0 mL.

## Results and discussion

Table 1 lists the various performance metrics for each bottle considering both phases (100 sips). The Mean Percentage Error (MPE) per sip, the Mean Absolute Error (MAE) per sip and the Cumulative MPE are calculated as follows:

$$\text{Sip} \; \text{MPE}=\frac{1}{n}\sum_{i=1}^{n}\frac{({S}_{act}^{i}- {S}_{est}^{i})}{{S}_{act}^{i}}\times 100$$

$$\text{Sip} \; \text{MAE}=\frac{1}{n}\sum_{i=1}^{n}\left|{S}_{act}^{i}- {S}_{est}^{i}\right|$$

$$\text{Cumulative} \; \text{MPE}=\frac{1}{n}\sum_{k=1}^{n}\frac{({C}_{act}^{k}- {C}_{est}^{k})}{{C}_{act}^{k}}\times 100$$

where ${S}_{act}^{i}$ and ${S}_{est}^{i}$ are the actual and estimated intake volume for ${i}_{th}$ sip, respectively and $n$ is the total number of sips. ${C}_{act}^{k}$ and ${C}_{est}^{k}$ represent the cumulative intake volume from the last $k$ sips. The Sip MPE looks at the percent error for each individual sip and the Cumulative MPE looks at the total percent error over time. According to the results in Table 1, the H2OPal has the minimum number of missed recordings, the lowest Sip MPE and the lowest Cumulative MPE. When determining the total intake over a period of time, the Mean Error is preferred as a comparative metric over the Mean Absolute Error (MAE). Because it accounts for the bottle’s ability to recover a poor measurement over time when recording the subsequent measurements. The sip MAE has also been included for applications where the accuracy of each sip is important, as it calculates the absolute error of each sip. The Cumulative MPE also measures how well the measurements balance out over the entire phase and does not penalize individual sips. Another observation was that 3 out of 4 bottles underestimated the volume intake per sip shown in Table 1 with negative numbers.

**Table 1:** Performance data for each commercial bottle.

Bottle type	# missed recordings	Mean error ± SD (ml)	Sip MPE (%)	Sip MAE (ml)	Cumulative MPE (%)	Correlation coefficient (r)
H2OPal	5	− 4.79 ± 21.66	− 2.10	12.78	1.9	0.88
HidrateSpark steel	7	− 6.17 ± 20.05	− 16.11	9.13	− 24.1	0.88
HidrateSpark 3	8	− 9.08 ± 15.24	− 14.9	13.26	− 13.3	0.94
Thermos Smart Lid	16	2.5 ± 35.43	14.64	23.84	2.1	0.75

The R-square Pearson correlation coefficients for all bottles are also shown in Table 1. The HidrateSpark 3 provided the highest correlation coefficient. Although the HidrateSpark 3 had some missed recordings, the majority of those were small sips (< 40 mL), so that it did not affect the correlation coefficient as heavily. The H2OPal and HidrateSpark Steel both had high correlations with r = 0.88, where the Thermos SmartLid had the lowest correlation (r = 0.75).

**Figure 2:**
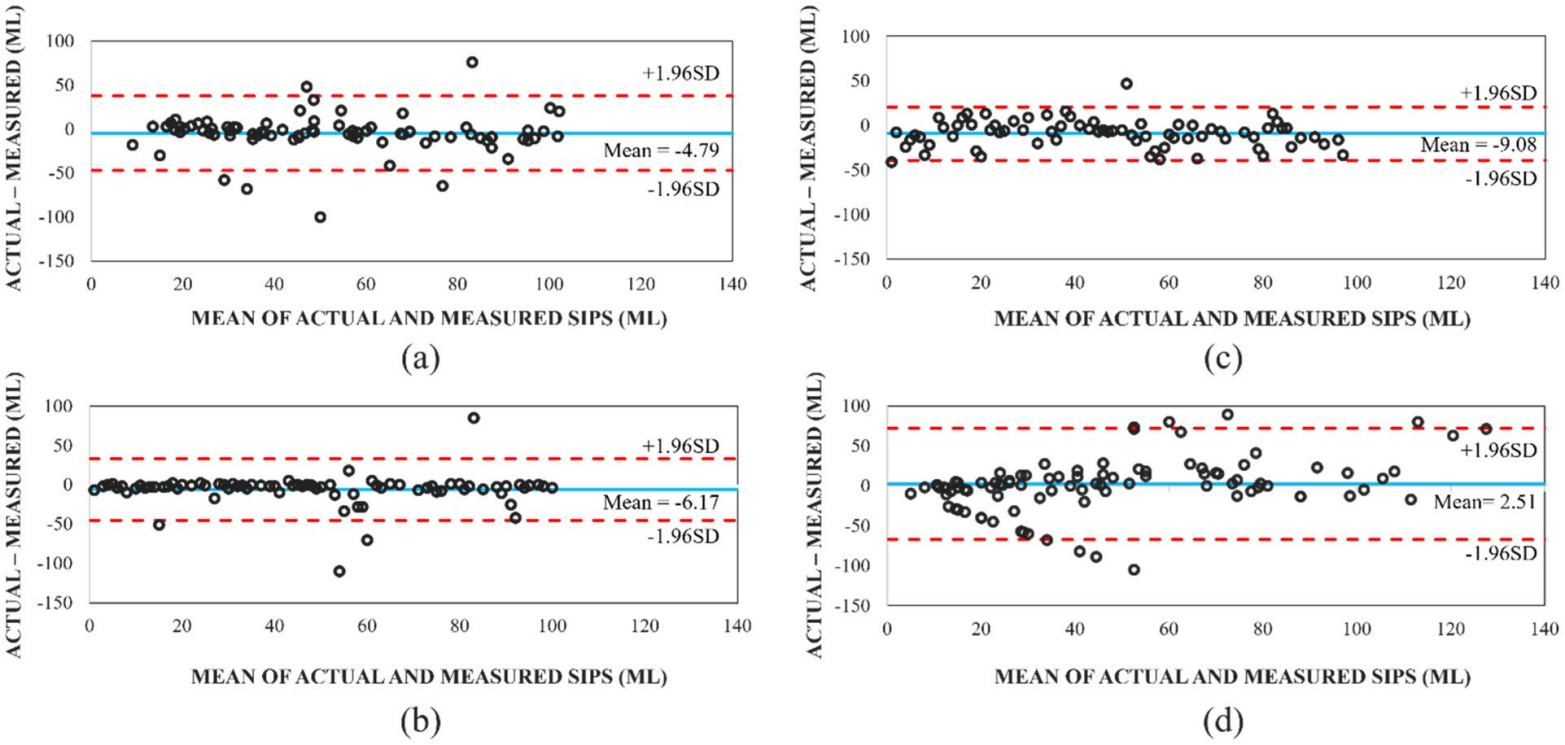
Bland–Altman plots for (**a**) H2OPal, (**b**) HidrateSpark Steel, (**c**) HidrateSpark 3, and (**d**) Thermos Smart Lid. The dotted lines represent the confidence intervals around the mean, calculated from the standard deviation in Table 1.

The Bland–Altman plots in Fig. 2 also confirmed that the HidrateSpark 3 had the smallest Limits of Agreements (LoA) compared to the other three bottles. The LoA analyzes the extent to which the actual and measured values agree. In addition, almost all measurements were in the range of LoAs confirming that this bottle provides consistent results, as shown in Fig. 2c. However, most of the values are below zero, meaning that generally the sip sizes are being underestimated. The same is true for the HidrateSpark Steel in Fig. 2b, where most of the error values are negative. Therefore, these two bottles provided the highest MPEs and Cumulative MPEs compared to H2Opal and Thermos Smart Lid where the errors were distributed above and below 0 as seen in Fig. 2a,d.

The HidrateSpark Steel and H2OPal had similar standard deviations of 20.04 mL and 21.41 mL, respectively. Figure 2a,b also demonstrated that the HidrateSpark Steel’s values bounced consistently around the mean but generally stayed within the LoA region, while the H2Opal had more values outside of the LoA region. The Thermos Smart Lid had the largest standard deviation of 35.42 mL and more than 10% of measurements were outside of the LoA region shown in Fig. 2d. This bottle provided the minimum Sip Mean Error and relatively small Cumulative MPE, despite having the highest number of missed recordings and largest standard deviation. The Thermos SmartLid had many missed recordings because the sensor straw does not extend to the bottom of the container, causing missed recordings when the liquid contents are below the sensor stick (around 80 mL). This should lead to underestimating the fluid intake; however, the Thermos is the only bottle that had a positive MPE and Sip Mean Error, meaning that the bottle is overestimating the fluid intake. Therefore, the reason the Thermos had a very low mean sip error is because nearly each measurement from the bottle is a large overestimation. When these overestimations are averaged including the many missed sips that are not recorded at all (or “underestimated”) the mean result balances out. When excluding the missed recordings from the calculation, the Sip Mean Error became + 10.38 mL, confirming that there is a large overestimation of individual sips. Though this may appear to be positive, in reality this bottle is inaccurate in individual sips estimation and unreliable as it misses many drinking events. Additionally, as seen in Fig. 2d, the Thermos SmartLid appears to have an increased error as the sip size increases.

In summary, the H2OPal is the most accurate at estimating sips over time and was the most reliable to measure most of the recordings. The Thermos Smart Lid was the least accurate and missed more sips than the other bottles. The HidrateSpark 3 bottles had more consistent error values, however underestimated the majority of sips leading to poor performance throughout time.

### Calibration

The results suggest that the bottles may have a certain offset that could be compensated using a calibration algorithm. This is especially true for the HidrateSpark bottles that have small standard deviations of errors and always underestimate individual sips. The Least-Square (LS) method was used with Phase 1 data while excluding any missed recordings to obtain the offset and gain values. The obtained equation was used on the measured sip intakes of Phase 2 to calculate the actual value and determine the error after calibration. Table 2 shows that the calibration improved the Sip Mean Error for both HidrateSpark bottles, but not the H2OPal or Thermos Smart Lid.

**Table 2:** Sip mean error before and after calibration for phase 2.

Bottle	Sip mean error before calibration (ml)	Sip mean error after calibration (ml)
H2OPal	− 2.65	− 3.02
HidrateSpark Steel	− **9.33**	− **6.15**
HidrateSpark 3	− **12.5**	− **10.25**
Thermos Smart Lid	− 0.125	− 7.18

Values that were improved after calibration are in bold.

### Bottle liquid level comparison

In Phase 1 to complete all the measurements, each bottle was refilled multiple times so it is possible that the calculated MAE is impacted by the filled level of the bottle. To determine this, each bottle was divided into three liquid levels as high, medium, and low, based on the total volume of each bottle. For the measurements in Phase 1, a one-way ANOVA test was performed to determine if the liquid level had a significant difference on the absolute error. For the HidrateSpark 3 and Steel, there was no significant differences in the error in each of the three categories. For the H2OPal and the Thermos bottles, there was a borderline significant difference (p < 0.05) using the Welsh test for unequal variance. Subsequently, a multiple comparison Tukey HSD test was performed on these two bottles. In both cases, the significant difference was between the “high” level and the “low” level categories. For the H2OPal, the “high” category had the largest mean and standard deviation, meaning that there is a higher error when the bottle is more filled. However, in the Thermos, the “low” category had the highest error. This is likely because the sensor does not extend to the bottom of the bottle.

### Real world vs simulated phases

A two tailed t-test was conducted to compare the Phase 1 and Phase 2 errors for each bottle. For all bottles, we achieved *p* > 0.05 meaning that the two populations were not significantly different. However, it was observed that the number of missed recordings was much greater in Phase 2 for both HidrateSpark bottles. For the H2OPal, the number of missed recordings were almost equal (2 vs 3), and for the Thermos SmartLid there were fewer missed recordings in free living scenario (6 vs 10). Since the HidrateSpark bottles were both improved after calibration, a t-test was also conducted after-calibration. For the HidrateSpark 3, the Phase 1 and 2 errors were borderline significantly different (p = 0.046). This is more likely due to the larger number of missed recordings in Phase 2 compared to Phase 1.

### Usability analysis and limitation

This section provides insight on the usability of the bottles and their apps, as well as additional functionality information. Although the accuracy of the bottles is important, the usability factors are also of interest when choosing a bottle.

#### App performance and functionality

The HidrateSpark 3 and HidrateSpark Steel are equipped with LEDs that blink to remind the users to drink if they are not on track to meet their goal, or to blink a certain number of times a day (set by the user). They can also be set to blink every time the user drinks. The H2OPal and Thermos Smart Lid do not have any visual feedback to remind the user to drink. However, all purchased bottles have mobile notifications to remind the users to drink via the mobile app. The number of notifications per day can be customized in the HidrateSpark and H2OPal app.

The HidrateSpark 3 and Steel use a linear trend to guide the user when to drink, and give an hourly suggested target the user should aim for to reach the goal by the end of the day. The H2OPal and Thermos Smart Lid only provide one total daily target. In all bottles, if the device is not connected to the app by Bluetooth, the data is stored locally and synchs once paired.

None of these four bottles focuses on elderly hydration. Additionally, the formulae the bottles use to determine the daily intake goal were not available, so it is difficult to determine if they are appropriate for seniors. The bottles are mostly large, heavy and are not tailored to seniors. The use of the mobile app may also not be ideal for seniors, though it could be useful for researchers to collect data, remotely.

#### Hardware and software limitations

All bottles could not determine if the liquid was consumed, discarded or spilled. All bottles also needed to be placed down on a surface after each sip to record the intake, accurately. This means that it is possibile to miss drinks if the bottle is not placed down, especially if it is refilled.

Another limitation is that the devices needed to be re-paired regularly with the app to synchronize the data. The Thermos needed to be re-paired every time the app is opened, and the HidrateSpark bottles often had difficulty finding the Bluetooth connection. The H2OPal was the easiest to re-pair with the app if the connection was interrupted. All bottles were calibrated before the testing began, and all had to be recalibrated at least once during the process. The HidrateSpark bottles and H2OPal had to be emptied and filled fully to calibrate.

All the bottles did not have the option to download or save data long term. Additionally, none were able to be accessed via an API.

#### Battery

The HidrateSpark 3 and H2OPal use replaceable lithium-ion batteries and the HidrateSpark Steel and Thermos SmartLid use rechargeable batteries. The rechargeable batteries should last up to 2 weeks on a full charge, as stated by the manufacturers, however, the Thermos SmartLid had to be charged almost every week when using it frequently. This is a limitation as many people will not remember to charge the bottles regularly.

#### Additional factors

There are various factors that impact the selection of a smart bottle especially when the users are elderly. The heaviness and bulkiness of the bottle is an important factor, as it needs to be easy to use for frail older adults. As mentioned, these bottles are not tailored to older adults. The price and the amount liquid each bottle can contain is also another factor. Table 3 shows the height, weight, liquid volume and price of each bottle. The Thermos Smart Lid is the cheapest and lightest, as it is made entirely of lighter plastic. It can also hold the most amount of liquid compared to other three bottles. Conversely, the H2OPal is the tallest, heaviest and most expensive among the study bottles.

**Table 3:** The height, weight, volume and price of the bottles.

Bottle	Height (cm)	Weight (g)	Liquid volume (oz)	Price (USD)
H2OPal	2	427.1	18.6	99.99
HidrateSpark Steel	23.5	410.8	17	64.99
HidrateSpark 3	26	329.2	20	59.95
Thermos Smart Lid	24	205.3	24	42.35

## Conclusion and future works

Commercially available smart bottles are very useful to researchers as there is no need to prototype a new device. Though there are many smart water bottles available, the most prevalent issue was that the data or raw signals were not accessible to the users, and only some had the results displayed in a mobile app. The development of a widely available smart bottle with high accuracy and completely accessible data is needed, especially one tailored to older adults. Of the four bottles tested, out of the box the H2OPal had the lowest Sip MPE, Cumulative MPE, and number of missed recordings. The HidrateSpark 3 had the highest linearity and smallest standard deviation and lowest MAE. The HidrateSpark Steel and HidrateSpark 3 can be simply calibrated manually to decrease the Sip Mean Error using LS method. For a more accurate sip recording, the HidrateSpark 3 is the preferred bottle, and for more consistent measurements over a period of time, the H2OPal is preferred. The Thermos SmartLid had the most unreliable performance with the most missed number of sips and a large over estimation of individual sips.

This study is not without limitations. In a real-world scenario, many users will drink from other vessels, especially for hot liquids, store bought beverages and alcohol. Future work should evaluate how the form factor of each bottle might affect the error to guide smart water bottle design.
